# Leicester Saturday Hospital Society

**Published:** 1920-04-03

**Authors:** 


### Leicester Saturday Hospital Society.

A RECORD CONTRIBUTION.

THE annual report of the Society has announced a record—an increase of £3,751 over the income of the previous year. The growth of the Society has been continuous, and each year since the Society was founded a handsome addition to the annual income has been made. The total sum contributed last year was £21,631. Sir Arthur Wheeler, Bart., has given a Convalescent Home for Children, which will shortly be opened with eighteen beds. The site is a high one with splendid views of the Charnwood Forest, and includes nearly three acres. The Desford Home for Men has been reopened after spring-cleaning and repairs necessitated by the military occupation. The Swithland Home for Women received 565 patients, and 216 members had been sent to other homes. The total expenditure was £7,468 8s. 5d.

The unanimous decision of the delegates to contribute twopence per week instead of one penny was partly responsible for the largely increased income, which will be still further evident this year. The report commended the enthusiasm and ability of Mr. H. H. Woolley, J.P., the Organising Secretary.

Mr. Sydney A. Gimson was elected President of the Society in place of the late Mr. J. Gipson Clarke, J.P.; Mr. W. Newbery and Mr. J. Holyoak were elected Vice-Presidents. The latter appointment is the first instance of a delegate having been appointed to this important office. Grants amounting to £120 were made to other institutions and to local Nursing Associations whose work was of help to the Society. Mr. T. Fielding Johnson, jun., J.P., Chairman of the Leicester Royal Infirmary, acknowledged the debt of that Institution to the Society's invaluable help.

